# Analysis of Animal Well-Being When Supplementing Drinking Water with Tramadol or Metamizole during Chronic Pancreatitis

**DOI:** 10.3390/ani10122306

**Published:** 2020-12-05

**Authors:** Guanglin Tang, Wiebke-Felicitas Nierath, Rupert Palme, Brigitte Vollmar, Dietmar Zechner

**Affiliations:** 1 Rudolf-Zenker-Institute of Experimental Surgery, Rostock University Medical Center, 18057 Rostock, Germany; guanglintang@hotmail.com (G.T.); Wiebke.Nierath@med.uni-rostock.de (W.-F.N.); brigitte.vollmar@uni-rostock.de (B.V.); 2 Unit of Physiology, Pathophysiology and Experimental Endocrinology, Department of Biomedical Sciences, University of Veterinary Medicine, 1210 Vienna, Austria; Rupert.Palme@vetmeduni.ac.at

**Keywords:** wellbeing, analgesia, rodents, sweetened water

## Abstract

**Simple Summary:**

Pain management during in vivo experiments can considerably improve the wellbeing of animals. However, often it is not clear, which drugs are best for the animals and how to apply these drugs without causing stress. In this study, we evaluated mice when metamizole or tramadol was provided via drinking water. Neither of these two drugs reduced the amount of consumed water or body weight in healthy mice or influenced their natural behavior, such as nest building or burrowing activity. Both analgesics were then given to mice suffering from chronic pancreatitis. Mice drinking tramadol supplemented water, at some time-points, experienced less loss in body weight and consumed more water than mice drinking metamizole. However, no major differences in other methods measuring wellbeing of mice was observed. In conclusion, both analgesics can be used during chronic pancreatitis, but tramadol seems to be moderately advantageous when compared to metamizole.

**Abstract:**

Pain management during in vivo experiments is an animal welfare concern and is in many countries also legally required. In this study, we evaluated C57Bl/6J mice when 3 g/L metamizole or 1 g/L tramadol was provided via drinking water, before and during cerulein-induced chronic pancreatitis. Supplementation of drinking water with metamizole or tramadol did not significantly reduce the amount of consumed water. In order to evaluate the wellbeing of mice, a distress score, burrowing activity, nesting behavior, and body weight was assessed. Before induction of pancreatitis, neither tramadol nor metamizole influenced these readout parameters. Chronic pancreatitis caused a significantly increased distress score, decreased burrowing activity and a reduction in body weight. Mice drinking tramadol-supplemented water experienced less loss in body weight and consumed more water than mice drinking metamizole, at a few time-points during chronic pancreatitis. Pancreatic atrophy, a characteristic feature of chronic pancreatitis was not differentially influenced by either analgesic. In conclusion, both analgesics can be used during 33 days of chronic pancreatitis, but tramadol seems to be moderately advantageous when compared to metamizole.

## 1. Introduction

Analgesia during potentially painful in vivo experiments is ethically required and in many countries legally obligatory as well [1,2,3]. Optimizing pain treatment for specific animal models will improve the well-being of animals and will thereby provide the basis for future research on pathophysiological processes and in vivo evaluation of drugs.

When deciding for appropriate analgesia, one problematic issue is the possible interference of analgesic treatment, with the studied pathology. For example, the non-steroidal-inflammatory-drug meloxicam is not reasonable for inflammation models, because of its anti-inflammatory property, due to its COX-2-inhibition [4]. Therefore, it is important to assess that analgesia does not interfere with the pathophysiology of a studied disease. For example, since it was demonstrated that metamizole does not interfere with progression of cerulein-induced acute pancreatitis in mice [5], some studies use metamizole as an analgesic when studying this disease in preclinical studies [6]. Metamizole is also often chosen to treat human pancreatitis patients [7,8], but other analgesics, such as tramadol are also used [9,10].

When evaluating, if analgesics reduce discomfort in mice, scientists often use readout parameters such as body weight loss, as well as reduced burrowing and nesting activity [11,12,13,14,15]. However, analgesics might also interfere with these readout parameters. For example, opioids, especially at a high concentration, were demonstrated to decrease burrowing activity and running activity, due to their sedative effect [16,17]. Additionally the opposite, increased locomotor activity after morphine administration was observed in some mouse strains [18]. Thus, interference with the readout parameters could falsify conclusions, when evaluating analgesics.

The application of analgesia is mostly done orally, subcutaneously, or via an intraperitoneal injection [19,20]. However, it is well-documented that injections and handling causes distress in rodents [21,22,23,24,25]. In order to minimize distress, scientists, therefore, strive for applying drugs via voluntary oral intake [26]. However, some substances are considered to be unpalatable [27,28]. Therefore, drinking water or food supplemented with analgesics are often sweetened [26,29,30,31,32].

One often used analgesic is metamizole, which is classified as a non-opioid, anti-pyretic pro-drug. It is soluble in water and suitable for oral administration [33]. After administration, it is hydrolyzed to 4-N-Methylaminoantipyrin (4-MAA). MAA reduces pain via working on the opioidergic and cannabinoid system, as well as blocking COX-3. In practice, it is used to reduce fever, neuropathic pain, and visceral pain [5,34,35,36]. For example, it reduces discomfort during acute pancreatitis in mice [5].

Tramadol is classified as a partial agonist on the opioidergic system and is FDA approved for the treatment of moderate to severe pain [37]. Like other opioids, tramadol intake potentially leads to abuse and addiction in humans [37,38,39]. Thus, non-opioid analgesics like metamizole are often preferred. Among various other pain conditions, tramadol is used for treating visceral pain, for example, in the endometriosis plus ureteral calculosis pain model or during gut inflammation [40,41].

The aim of this study was to compare metamizole and tramadol for improving the wellbeing of mice during animal experiments. We focused on chronic pancreatitis, which is an acknowledged painful disease in humans [42,43,44]. For this purpose, we studied C57Bl/6J mice, before and during repetitive cerulein injections.

## 2. Material and Methods

### 2.1. Animals

These animal experiments were conducted in accordance with the European Union Directive 2010/63/EU and German law for animal protection (TierSchG). The public authority (Landesamt für Landwirtschaft, Lebensmittelsicherheit und Fischerei Mecklenburg-Vorpommern, 7221.3-1-002/17) and the §15 (according to the TierSchG) committee of Mecklenburg-Vorpommern, approved all experiments and experimental protocols. The mice were housed in type III cages at 12-h dark light cycle, constant temperature (21 ± 2 °C) and humidity (60 ± 20%). Autoclaved bedding (Bedding Espe Max 3–5 mm granulate, H 0234-500, Abedd, Vienna, Austria) was used, and water and food (pellets, V1534.000, 10 mm, ssniff Spezialdiaeten GmbH, Soest, Germany) were provided ad libitum. Enrichment was provided by a fun tunnel (75 × 38 mm paper tunnel, H 0528-151, ssniff Spezialdiaeten GmbH), nesting material (Zoonlab GmbH, Castrop-Rauxel, Germany), and a wooden block (Espe size S, 40 × 16 × 10 mm, H0234.NSG, Abedd, manufacturer, Vienna, Austria). 

**Figure 1:**
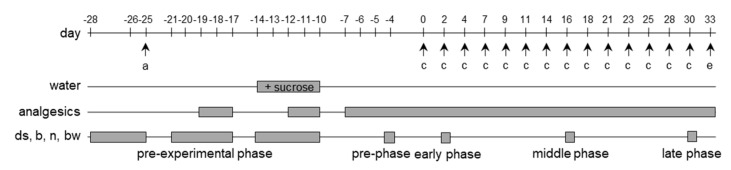
Experimental procedure for evaluating analgesics. C57Bl/6J mice were allocated (a) to a metamizole or a tramadol group on day −25 and these analgesics were added to the drinking water as indicated. The water was supplemented with sucrose on day −14 to day −10. Chronic pancreatitis was induced by three repetitive cerulein injections (c) at the indicated days, and the mice were euthanized (e) on day 33 in order to analyze the pancreas. A distress score (ds), burrowing (b), as well as nesting (n) activity and the body weight (bw) was evaluated during the indicated phases of the experiment. metamizole: *n* = 7; tramadol: *n* = 7.

14 C57Bl/6J mice, which were 107 to 113 days old (at day 0 of the experimental schema) were used for this study. They were allocated in a non-random manner either to a metamizole (7 mice) or a tramadol (7 mice) group, because we matched their median burrowing activity on day −25, in order to distribute the burrowing activity of mice evenly between these two groups. We used surplus male mice for this study, because the female mice were required to expand the mouse strain. Please note that the focus on male mice might be a limitation of this study. In order to minimize the number of animals used for this study, this study was stopped at a group size of *n* = 7. In this study, fecal corticosterone metabolites were evaluated in a blinded manner. Due to the weak yellow color of metamizole containing drinking water, the observer was not completely blinded when evaluating all other readout parameters. No animals were excluded from analysis or had to be euthanized, because they reached a humane endpoint. Humane endpoints were defined in our previously published distress score (body weight loss of −20%, a single distress score of 5 or a total distress score of >15) [45]. In order to evaluate, if analgesics influence animal behavior, or for pain relief, 3 g/L metamizole (Ratiopharm, Ulm, Germany) or 1 g/L tramadol (Ratiopharm) was provided in the drinking water (Figure 1). Fresh drinking water was prepared daily. We also wanted to address the question in the pre-experimental phase, if adding sucrose to the drinking water leads to a higher water consumption. As the C57Bl/6J mouse strain was reported to be especially susceptible to sweetened water when compared to other mouse strains [46,47], sucrose was added at a low concentration of 0.5% to the drinking water on day −14 to day −10 [48].

Chronic pancreatitis was induced in these mice with cerulein (Bachem H-3220.0005, Bubendorf, Switzerland) dissolved in 0.9% sodium chloride. It was administered by consecutive intraperitoneal (i.p.) injections (50 μg/kg, three hourly injections/day; three days/week (on day 0, 2, 4, 7, 9, 11, 14, 16, 18, 21, 23, 25, 28, and 30) [6]. The last injection was between 14:30–15:00. The mice were euthanized on day 33 under isoflurane anesthesia, through cervical dislocation, and the pancreas to body weight ratio of each mouse was recorded.

### 2.2. Analysis of Animal Distress and Water Consumption

Before the actual experiment, the mice were housed in groups of 3–5 mice and were allowed to burrow and nest twice (day 35 to 31, before the first pancreatitis induction). Starting on day −31, each mouse was housed in a separate cage and the distress score, burrowing activity, and nesting activity was evaluated on the days, as indicated in each figure. The body weight was determined 24 h after the indicated days, in order to allow changes in body weight in response to experienced distress (e.g., the cerulein injections). During the identical 24 h period, feces was collected from home cages, dried for 4 h at 65 °C, and stored at −20 °C. Dry feces was then extracted with 80% methanol for subsequent analysis of fecal corticosterone metabolites, using a well-validated 5α-pregnane-3β,11β,21-triol-20-one enzyme immunoassay, as previously published [49,50,51]. An overview of distress evaluation is presented in Figure 1.

The distress-score was based on other score sheets [52,53] and previously published by our working group [45,49,54,55]. The score evaluates the general condition, spontaneous behavior, and flight behavior of mice, and was assessed 30 min after the last injection of cerulein (15.00–15.30). During chronic pancreatitis on 4 out of 7 tramadol-treated and 4 out of 7 metamizole-treated animals, ruffled fur (piloerection) was noticed at day 2.

Burrowing activity was quantified by using a burrowing tube (15 cm length × 6.5 cm diameter), which was filled with 200 ± 1 g food pellets (ssniff Spezialdiaeten GmbH; the animals had access to these pellets (in addition to food placed in their rack). One tube was put in the cage two and a half to three hours before the dark phase and the other was placed to one and a half hour after the last cerulein injection (16:30–17:00). The weight of the food pellets (g) left in the tube was measured and deducted from 200 g after two hours.

In order to analyze nest building behavior, a cotton nestlet (5 cm square of pressed cotton batting, Zoonlab GmbH, Castrop-Rauxel, Germany) was placed in the cage 0–30 min before the dark phase (at 18:30–19:00). Pictures of the nests were taken and nesting was scored at the end of the dark phase, following a standard operating procedure by a single observer, or independently by two observers (in those cases where scoring was difficult for the first observer). The nests were evaluated, by modifying a scoring system developed by Deacon [56] (adding a 6th score point defining a perfect nest—the nest looks like a crater and more than 90% of the circumference of the nest wall was higher than the body height of the coiled up mouse).

In order to assess water consumption, the weight of the water bottle was measured on the days as indicated in the relevant figures (about 12:00 a.m.) and weighted again 24 h later. The difference in weight was calculated in milliliter (1 g of water was considered to be 1 mL of volume) and divided by the body weight of the animal (measured in g).

### 2.3. Graphs and Statistical Analysis

Graphs show single data points, median, and interquartile range. Data were graphed and all biostatistical analysis was done using the GraphPad Prism8 (GraphPad Software Inc., San Diego, CA, USA). Statistical significance was determined by different methods (for details see each figure legend) based on the number of independent variables and data characteristics. If the influence of two independent variables such as time and analgesics were evaluated on one dependent variable, a two-way repeated measure ANOVA with Geisser–Greenhouse correction was performed. In case the water bottles leaked and therefore some data were missing, a mixed-effect model was used for the two-way repeated measure ANOVA (for details see figure legends). If the influence of one independent variable on a dependent variable was evaluated, the normality of data was evaluated by the Shapiro–Wilk normality test. In case of normality, the paired *t*-test and in case of failed normality the Wilcoxon signed-rank test was performed, when the data were paired (e.g., identical mice at different time points). When the data were not paired, the unpaired *t*-test (in case of normality of data) or the Mann–Whitney rank sum test (in case of failed normality) was performed. When analyzing the pancreas weight to body weight ratio of the three groups, a one-way ANOVA was used, since the data passed the Shapiro–Wilk normality test. Appropriate methods for correction of multiple comparisons were performed, as suggested by the software (see figure legends for details). Differences with *p* ≤ 0.05 were considered to be significant.

## 3. Results

**Figure 2:**
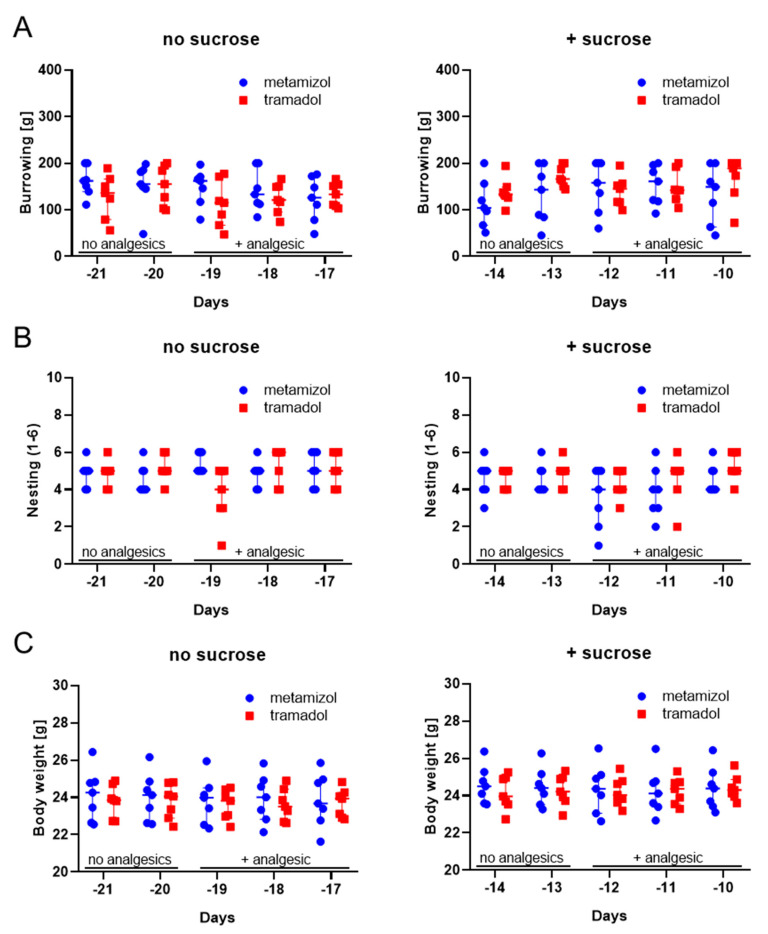
Impact of analgesics on burrowing as well as nesting activity and body weight during the pre-experimental phase. Burrowing activity (**A**), nesting activity (**B**), and percentage of body weight compared to day −26 (**C**) was assessed, using drinking water without or with sucrose. No statistically significant differences between metamizole and tramadol treatment were obtained (using two-way repeated measure ANOVA with Sidak’s correction for multiple comparisons). Metamizole: *n* = 7; tramadol: *n* = 7.

**Figure 3:**
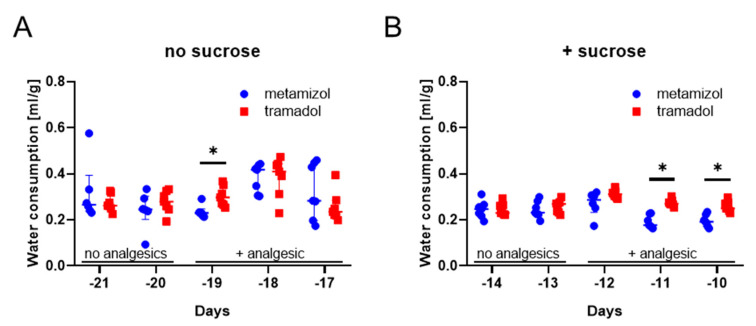
Impact of analgesics on water consumption during the pre-experimental phase. Water Consumption (mL) divided by body weight (g) was assessed on the days indicated, using drinking water, without (**A**) or with sucrose (**B**). * Statistically significant differences between metamizole and tramadol treatment was obtained (using two-way repeated measure ANOVA in a mixed-effect model with Sidak’s correction for multiple comparisons). Metamizole: *n* = 6–7 (since the water bottle for one animal leaked on day −21, day −20, day −19, and day −12, these data were not included); tramadol: *n* = 7.

Before evaluating metamizole and tramadol during chronic pancreatitis, we verified that these analgesics did not influence our readout parameters for animal distress. Therefore, we tested in the pre-experimental phase, if these analgesics did influence the distress score, burrowing activity, nesting behavior or the body weight. We supplied metamizole or tramadol to the drinking water (for experimental design see Figure 1). Independent of sucrose supplementation, the two analgesics did not have a significant influence on burrowing activity, nesting behavior, or the body weight of mice during the pre-experimental phase (Figure 2A–C). We also evaluated, if these analgesics influence water consumption. Neither metamizole nor tramadol caused a reduction in water consumption (Figure 3A). When adding sucrose, again a similar amount of water was drunk by the animals, irrespective of whether metamizole or tramadol was added (Figure 3B). In water without (Figure 3A) and with sucrose (Figure 3B), mice consumed more tramadol-containing water at some time-points, when compared to metamizole-containing water. In addition, we evaluated the influence of sucrose to water consumption. Sucrose did not increase consumption of water (Supplementary File 1). When added to water containing analgesics, it actually decreased water consumption of metamizole or tramadol (Supplementary File 1). Therefore, no sucrose was added to the drinking water, during chronic pancreatitis.

**Figure 4:**
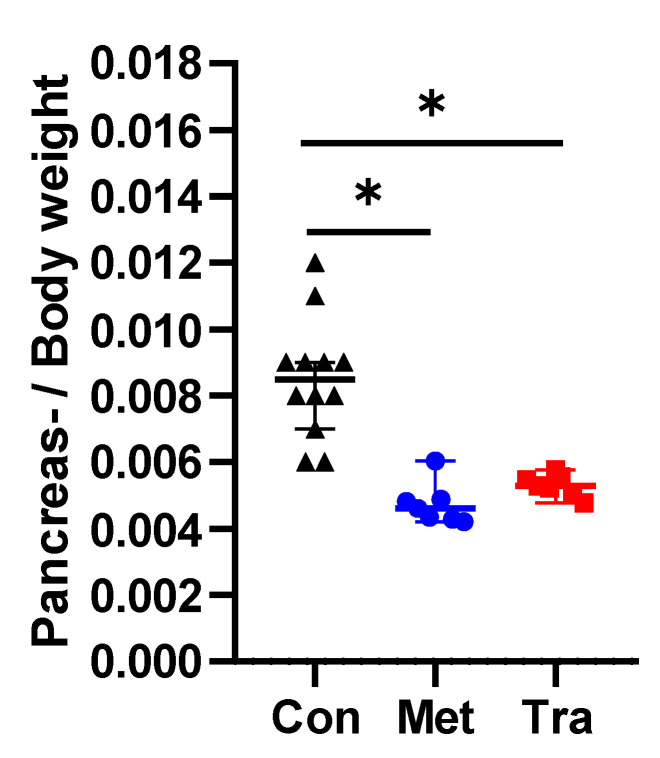
Pancreas weight to body weight ratio without and after chronic pancreatitis. The pancreas weight to body weight ratio of healthy mice (Con, black triangles) was significantly higher than the ratio observed in mice, which suffered from chronic pancreatitis and drank metamizole (Met, blue circles) or tramadol (Tra, red squares). No significant difference between the metamizole- and tramadol-treated animals was observed. * *p* ≤ 0.05 (one-way ANOVA with Sidak’s correction for multiple comparisons). Healthy mice: *n* = 12, metamizole: *n* = 7; tramadol: *n* = 7.

We first examined, if the choice of analgesic had an influence on the severity of chronic pancreatitis. We characterized atrophy of the pancreas by comparing the pancreas weight to body weight ratio. Significantly lower ratios were obtained in mice, which suffered from chronic pancreatitis, when compared to healthy mice (Figure 4). However, no significantly different ratios were observed when comparing mice treated with metamizole to mice treated with tramadol (Figure 4). This demonstrates that treatment with cerulein caused atrophy of the pancreas, a typical feature of chronic pancreatitis. Moreover, it points out that the choice of analgesic had no obvious influence on the severity of pancreatic atrophy.

**Figure 5:**
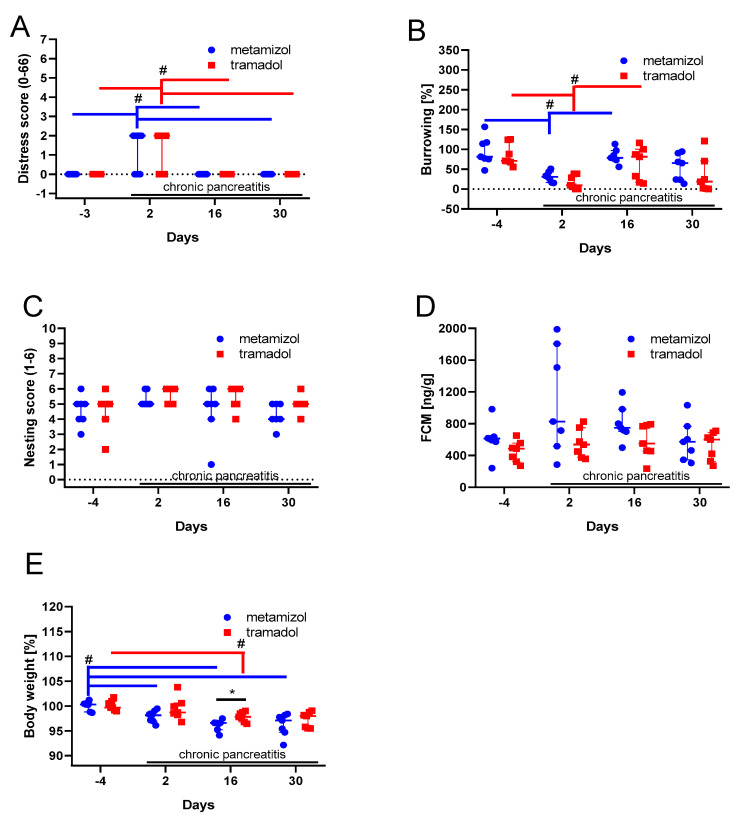
Impact of analgesics on readout parameters for animal distress during chronic pancreatitis. A distress score (**A**), the percentage burrowing activity compared to day −5 (**B**), nesting activity (**C**), fecal corticosterone metabolites concentration (**D**) and the percentage of body weight compared to day −5 (**E**) was assessed before (day −4) and during the early (day 2), middle (day 16), and late phase (day 30) of chronic pancreatitis. ^#^ *p* ≤ 0.05 between indicated days (two-way repeated measure ANOVA with Tukey correction for multiple comparison). * *p* ≤ 0.05 between the metamizole and the tramadol groups were obtained (two-way repeated measure ANOVA with Sidak’s correction for multiple comparisons). Metamizole: *n* = 7; tramadol: *n* = 7.

**Figure 6:**
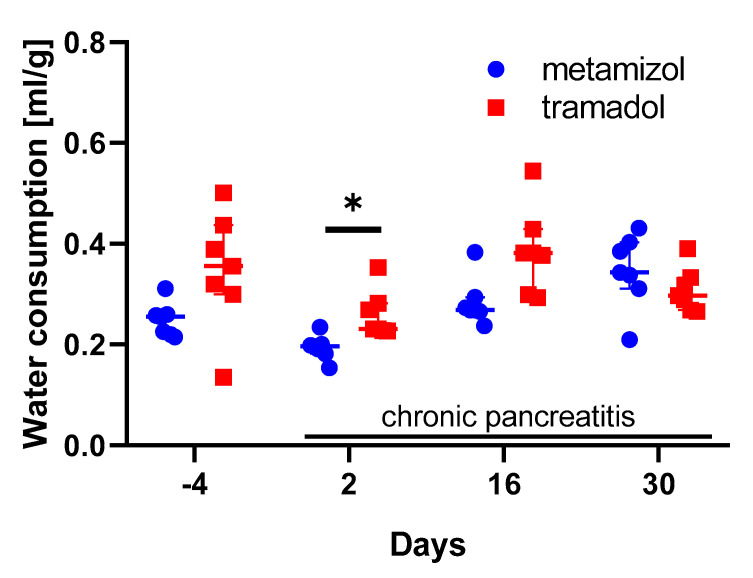
Impact of analgesics on water consumption during chronic pancreatitis. Water consumption (mL) divided by body weight (g) was assessed before (day −4) and during the early (day 2), middle (day 16), and late phase (day 30) of chronic pancreatitis. * *p* ≤ 0.05 between the metamizole and tramadol group was obtained (two-way repeated measure ANOVA with Sidak’s correction for multiple comparisons). Metamizole: *n* = 7; tramadol: *n* = 7.

Then, the distress of mice was evaluated during chronic pancreatitis, by assessing the distress score, burrowing activity, nesting behavior, fecal corticosterone metabolites concentration, and body weight change of these mice. The distress score was significantly increased in metamizole, as well as the tramadol-treated animals on day 2, during the early phase of chronic pancreatitis, but not at later phases of chronic pancreatitis (Figure 5A). Consistent with this observation, the burrowing activity was significantly decreased during the early phase of chronic pancreatitis, but recovered during the later phases (Figure 5B). Neither nesting activity (Figure 5C), nor concentrations of fecal corticosterone metabolites (Figure 5D) was significantly changed by chronic pancreatitis. The body weight was significantly reduced in metamizole-treated animals during all phases of chronic pancreatitis (day 2, day 16, and day 30), when compared to the pre-phase (Figure 5E). In the tramadol-treated animals, the body weight was significantly reduced during the middle phase (Figure 5E). The reduction in body weight was less severe in the tramadol-treated animals when compared to the metamizole-treated animals (Figure 5E). The analysis of water consumption demonstrated that during the early phase of chronic pancreatitis, more tramadol-supplemented water was consumed than metamizole-supplemented water (Figure 6). Thus, mice consumed more tramadol containing water during the early phase of chronic pancreatitis and had significantly higher body weight during the middle phase of pancreatitis.

## 4. Discussion

This study demonstrated that supplementing drinking water with tramadol is moderately advantageous, when compared to metamizole in a mouse model for chronic pancreatitis (Figure 5 and Figure 6). This conclusion is based on less body weight loss and higher fluid intake when tramadol was added to the drinking water (Figure 5 and Figure 6). Please note that the study is based on a group size of *n* = 7 and that possibly, at higher group sizes, additional significant differences between the metamizole group and the tramadol group might be observed. To support this main conclusion, it was important to verify that these drugs did not interfere with read-out parameters for animal wellbeing. Indeed, no major influence of these drugs on the distress score, burrowing activity, nesting behavior or body weight of healthy mice was observed (Figure 2). An interesting side aspect of this study was the observation that adding metamizole or tramadol to drinking water did not reduce water consumption (Figure 3A). Consistent with this observation, this study also demonstrated that supplementing drinking water with sucrose did not increase fluid intake (Supplementary File 1). 

Sweetening of non-palatable drugs is often necessary in order to facilitate that rodents eat or drink them. For example, this was published for the antiparasitic drug albendazol [57]. Thus, in many publications drugs were supplemented with a sweet nut paste, honey, or sucrose, to encourage mice to voluntarily consume drugs [31,58,59]. Even commercially available palatable delivery systems such as MediGel or Syrspend SF were tested for that purpose [11,29]. Sweeteners were also used, for example, to mask the bad taste of buprenorphine [58]. It was also suggested that water should be sweetened when adding tramadol to the drinking water [60]. In this study, we could not observe that mice consumed significantly less water when it was supplemented with tramadol or metamizole (Figure 3). Consistent with this observation, adding sucrose did not also increase the consumption of metamizole or tramadol (Supplementary File 1). We concluded that tramadol and metamizole were palatable to C57BL/6J mice, so that adding sucrose was not necessary. A warning that water is too often sweetened during animal experiments was also published previously with respect to the induction of the Tet-On system, with doxycycline [59].

We also observed that adding sucrose to the drinking water, in the absence of analgesic drugs did not increase water consumption (Supplementary File 1A,E). This seems to contradict publications that demonstrated that mice prefer to drink sweetened water [48,61,62]. The C57Bl/6J mouse strain was reported to be especially susceptible to sweetened water, when compared to other mouse strains [46,47]. However, all these publications describe sucrose preference tests. During such a test, the mouse can choose between two or more drinking options, whereas in our experiments, the mice always just had one bottle with drinking water either sweetened or not sweetened. We conclude that C57Bl/6J mice, when offered a choice, prefer sweetened water. However, sweetening of water does not necessarily encourage them to consume more than what is needed for quenching their thirst. However, we want to emphasize that we used only 0.5% sucrose and we, therefore, cannot exclude the possibility that mice would drink significantly more water when higher concentrations of sucrose were used. This is a limitation of this aspect of the study, even when we consider that C57Bl/6J mice are very sensitive to low concentrations of sucrose [48].

It was the main focus of this study to address the question, which analgesic is advantageous in improving animal welfare during chronic pancreatitis. As an important first step, we assessed if tramadol or metamizole influence the readout parameters, which were used for evaluating the distress of mice. It was previously reported that analgesics can influence such readout parameters, even in the absence of pain induction. For example, buprenorphine increases locomotor activity in mice at low doses [63] and causes sedation at high concentration [64]. In our experiments, neither the distress score, nor burrowing, as well as nesting activity or the body weight was significantly altered when adding either metamizole or tramadol to the drinking water (Figure 2). This supports the concept that no direct effect of these analgesics on these readout parameters interfered with the assessment of chronic pancreatitis. 

When comparing metamizole to tramadol during chronic pancreatitis, no significant differences in the distress score, burrowing, or nesting activity were observed (Figure 5). Nevertheless, there seems to be a small advantage to use tramadol as an analgesic, because mice consume more tramadol water during the early phase of pancreatitis and experience less loss in body weight during the middle phase of pancreatitis, when compared to mice drinking metamizole (Figure 5E and Figure 6). The following assumptions can explain these two observations. Possibly, tramadol was slightly more effective in reducing discomfort than metamizole, which results in higher consumption of water during the early phase and reduced body weight loss during the middle phase of chronic pancreatitis. However, in the late phase of chronic pancreatitis, tramadol was not beneficial any more. Maybe the mice develop tolerance towards this opioid. The hypothesis that tramadol can be more effective in reducing pain than metamizole is supported by a publication evaluating pain-induced functional impairment, after injecting uric acid in the knee joints of rats [65]. This concept is also supported by clinical studies, for example, for analgesia after ambulatory hand surgery [66]. However, also the opposite, that metamizole is more effective than tramadol was observed in arthritic rats and humans, e.g., in colic pain [67,68,69]. In addition, some studies did not detect a significant difference between these two analgesics [70]. The hypothesis that mice can develop tolerance towards tramadol is supported by studies using hot plate tests [71]. However, tramadol causes less tolerance than other opioid analgesics, such as morphine in rodents as well as humans [72,73].

## 5. Conclusions

In conclusion, this publication suggests that metamizole or tramadol are safe for mice, when supplied in drinking water continuously for 33 days, during chronic pancreatitis. In addition, no difference in pancreas to body weight ratio was noticed, suggesting that these analgesics did not influence the progression of chronic pancreatitis differently. Only at few time-points, two out of five parameters (body weight and water consumption) suggest that mice experience less distress when they are drinking tramadol instead of metamizole-supplemented water. Thus, drinking tramadol instead of metamizole-containing water provides only a moderate but not a major advantage during chronic pancreatitis.

## Supplementary Materials

The following are available online at https://www.mdpi.com/2076-2615/10/12/2306/s1, Supplementary File 1: Impact of sucrose on water consumption. Supplementary File 2: Raw data.
