# Supplementary material for: Analysis of Animal Well-Being When Supplementing Drinking Water with Tramadol or Metamizole during Chronic Pancreatitis
Source: Animals (Basel). 2020 Dec 5;10(12):2306. doi: 10.3390/ani10122306 (PMC7762076; doi:10.3390/ani10122306)
Supplement: Supplementary file 1 [file animals-10-02306-s001.zip › Supplementary File 1-2020-12-3.pdf]

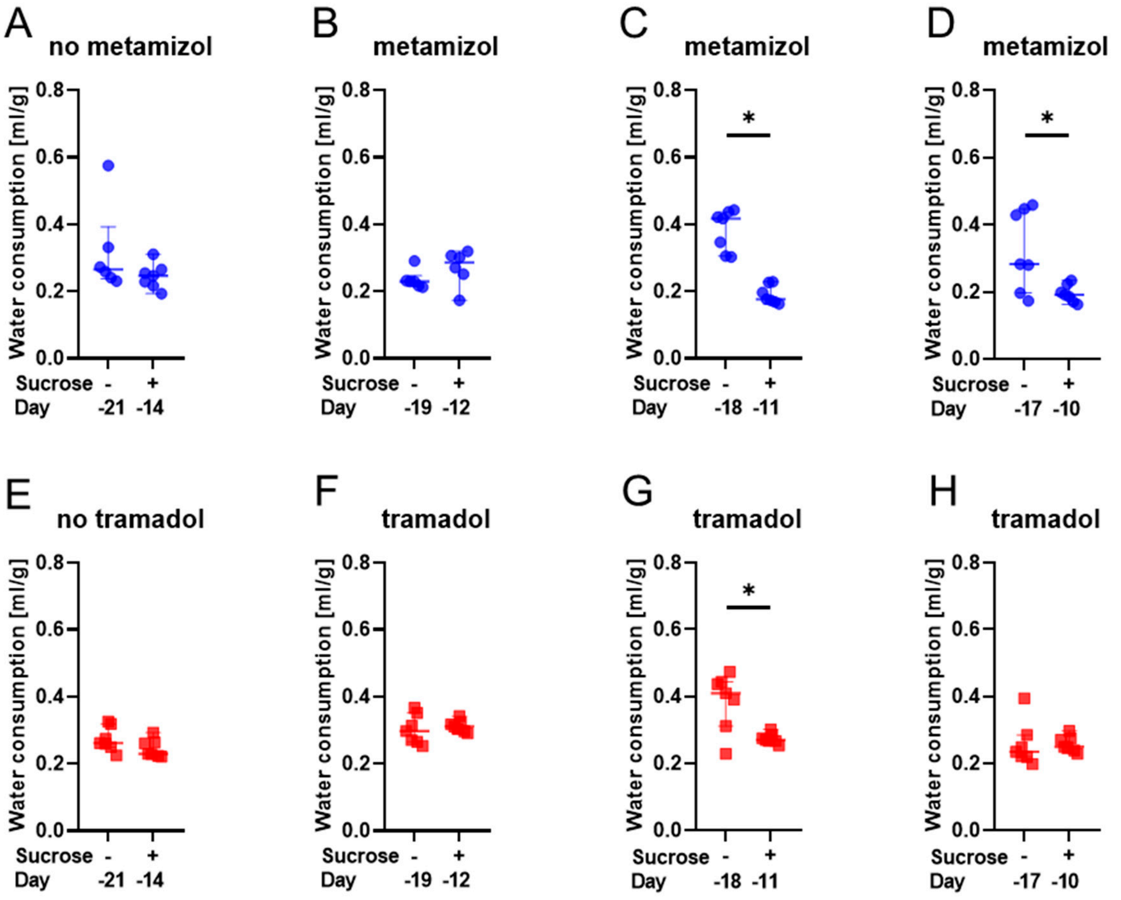

Supplementary File 1. Impact of sucrose on water consumption during the pre-experimental phase. Consumption of drinking water (ml) divided by body weight (g) in the metamizol (A, B, C, D) or tramadol (E, F, G. H) group was assessed, using drinking water without (day -21, -19, -18, -17) or with sucrose (day -14, -12, -11, -10). *Statistically significant differences between sucrose and no sucrose were obtained (normality of data: paired t-test test). Metamizol (blue circles): n = 6-7 (since the bottle of one animal leaked on day -21, day -19, and day -12, these data were not included); tramadol (red squares): n = 7.
